# Supplementary material for: A reappraisal of Theroteinus (Haramiyida, Mammaliaformes) from the Upper Triassic of Saint-Nicolas-de-Port (France)
Source: PeerJ. 2016 Oct 19;4:e2592. doi: 10.7717/peerj.2592 (PMC5075691; doi:10.7717/peerj.2592)
Supplement: Supplemental Information 1 [file peerj-04-2592-s001.docx]

| Material | Voxel size (mm) | Voltage (kV) | Current (A) |
| --- | --- | --- | --- |
| MNHN.F.SNP 2 Ma | 0.00328108 | 75 | 200 |
| MNHN.F.SNP 61 W | 0.00316999 | 60 | 240 |
| MNHN.F.SNP 78 W | 0.00303976 | 60 | 240 |
| MNHN.F.SNP 226 W | 0.00328108 | 75 | 200 |
| MNHN.F.SNP 309 W | 0.00316999 | 60 | 240 |
| MNHN.F.SNP 366 W | 0.00316999 | 60 | 240 |
| MNHN.F.SNP 487 W | 0.00303976 | 60 | 240 |
| MNHN.F.SNP 497 W | 0.00316999 | 60 | 240 |
| MNHN.F.SNP 722 | 0.00328108 | 75 | 200 |
