# Supplementary material for: A reappraisal of Theroteinus (Haramiyida, Mammaliaformes) from the Upper Triassic of Saint-Nicolas-de-Port (France)
Source: PeerJ. 2016 Oct 19;4:e2592. doi: 10.7717/peerj.2592 (PMC5075691; doi:10.7717/peerj.2592)
Supplement: Supplemental Information 2 — The null hypothesis is “population is normally distributed.” Since no p-value is lower than the threshold of 0.005, the null hypothesis cannot be rejected. Consequently, all sets of data are considered as normally distributed. [file peerj-04-2592-s002.docx]

| Set of data | numbers | Value of the test (W) | p-value |
| --- | --- | --- | --- |
| *T.nikolai* length | 5 | 0.8928 | 0.3714 |
| *T.nikolai* width | 5 | 0.9403 | 0.6684 |
| *T. nikolai* length/width | 5 | 0.9036 | 0.4304 |
| *T. rosieriensis* length | 7 | 0.9552 | 0.7769 |
| *T. rosieriensis* widht | 8 | 0.9632 | 0.8397 |
| *T. rosieriensis* length/width | 7 | 0.9946 | 0.9988 |
